# Negative life events and stress sensitivity in youth’s daily life: an ecological momentary assessment study

**DOI:** 10.1007/s00127-022-02276-0

**Published:** 2022-04-25

**Authors:** Christian Rauschenberg, Julia C. C. Schulte-Strathaus, Jim van Os, Matthieu Goedhart, Jan N. M. Schieveld, Ulrich Reininghaus

**Affiliations:** 1 Department of Psychiatry and Neuropsychology, School for Mental Health and Neuroscience, Maastricht University, Maastricht, The Netherlands; 2 Department of Public Mental Health, Central Institute of Mental Health, Medical Faculty Mannheim, University of Heidelberg, J5, 68159 Mannheim, Germany; 3 Department of Psychiatry, Brain Centre Rudolf Magnus, University Medical Centre Utrecht, Utrecht, The Netherlands; 4 Psychosis Studies Department, Institute of Psychiatry, Psychology and Neuroscience, King’s College London, London, UK; 5 Tilburg School of Humanities, Tilburg University, Tilburg, The Netherlands; 6 Mutsaers Foundation and Educational Institute Wijnberg, Venlo, The Netherlands; 7 Division of Child and Adolescent Psychiatry, Department of Psychiatry and Psychology, Maastricht University Medical Centre (MUMC+), Maastricht, The Netherlands; 8 ESRC Centre for Society and Mental Health, King’s College London, London, UK; 9 Centre for Epidemiology and Public Health, Health Service and Population Research Department, Institute of Psychiatry, Psychology and Neuroscience, King’s College London, London, UK

**Keywords:** Life events, Stress sensitivity, Youth mental health, Psychopathology, EMA

## Abstract

**Purpose:**

Negative life events (LEs) are associated with mental health problems in youth. However, little is known about underlying mechanisms. The aim of the study was to investigate whether exposure to LEs modifies stress sensitivity in youth’s daily life.

**Methods:**

Ecological Momentary Assessment (EMA) was used to assess stress sensitivity (i.e., association of momentary stress with (i) negative affect and (ii) psychotic experiences) in 99 adolescents and young adults (42 service users, 17 siblings, and 40 controls; *M*_age_ 15 years). Before EMA, exposure to LEs (e.g., intrusive threats, experience of loss, serious illness) was assessed.

**Results:**

Lifetime as well as previous-year exposure to LEs modified stress sensitivity in service users: they experienced more intense negative affect and psychotic experiences in response to stress when high vs*.* low exposure levels were compared. In contrast, controls showed no differences in stress sensitivity by exposure levels. Looking at specific types of LEs, controls showed less intense negative affect in response to stress when high vs*.* low exposure levels to threatening events during the last year, but not lifetime exposure, were compared. In siblings, no evidence was found that LEs modified stress sensitivity.

**Conclusion:**

Stress sensitivity may constitute a putative risk mechanism linking LEs and mental health in help-seeking youth, while unfavourable effects of LEs on stress sensitivity may attenuate over time or do not occur in controls and siblings. Targeting individuals’ sensitivity to stress in daily life using novel digital interventions may be a promising approach towards improving youth mental health.

**Supplementary Information:**

The online version contains supplementary material available at 10.1007/s00127-022-02276-0.

## Introduction

Most mental disorders manifest early (75% by age 24) and often increase in severity and specificity over time [1]. The onset of many mental disorders—e.g., psychotic, anxiety, mood, personality, eating, and substance-use disorders—fall into discrete time periods spanning from early adolescence (before age 14) to early adulthood (before age 25) [2]. Consequently, there has been a reform of youth mental health services [3] aimed at disrupting illness trajectories at developmentally early stages [4]. However, development and implementation of early intervention strategies are complicated by high comorbidity rates [2], and limited knowledge of underlying mechanisms, especially in the realm of youth mental health.

Mental health problems (e.g., depression, anxiety, psychosis) frequently co-occur during early stages of psychopathology [4] and share socio-environmental (e.g., negative life events) and genetic risk. This supports the notion of transdiagnostic phenotypes, including the extended psychosis spectrum phenotype [5], which are characterized by temporal and phenomenological continuity across developmental stages of clinical and subclinical mental health problems crossing traditional diagnostic boundaries. For instance, individuals reporting psychotic experiences are at increased risk of developing psychotic and affective disorders and the presence of psychotic experiences has been shown to predict greater illness severity as well as poorer treatment outcomes [5]. Thus, psychotic experiences may represent a severity marker of psychopathology [5], and subclinical as well as clinical expressions of affective dysregulation and psychosis may help elucidate putative underlying mechanisms through which socio-environmental risk factors, such as negative life events, impact on poor mental health.

Life events (LEs) are situations with clear beginnings and endings that generate positive or negative changes within personal circumstances, and/or contain an element of immediate threat [6]. Widely studied LEs include exposure to serious illness, death of a family member, financial hardship, intrafamilial conflict, relationship conflicts and divorce, occupational changes, and legal problems [6]. Increasing evidence suggests exposure to LEs is, as other forms of adverse experiences, non-specifically associated with psychopathology [6–8] such as depression [9], schizophrenia [10], anxiety [11], attention-deficit/hyperactivity disorder [12], and suicidality [13]. Moreover, studies suggest a dose–response relationship in which higher numbers of LEs and the co-occurrence with other socio-environmental risk factors are associated with increased severity of psychopathology, including psychosis [14]. This is in accordance with a study demonstrating that individuals with first-episode psychosis were almost four times more likely than healthy controls to have encountered both childhood and recent stressful LEs (e.g., death, divorce, sickness/accidents), in contrast to exposure to early LEs alone [15]. Considering that adverse childhood experiences (ACEs) are linked to later psychopathology [2], and an increasing number of LEs exposure is suggested to increase odds of developing various mental health problems, including first-episode psychosis [14], it is crucial to investigate LEs in the context of early-stage psychopathology.

Studies have also investigated associations of *specific types* of LEs with mental health problems. For instance, LeMoult et al. (2019) have shown that the death of a family member was associated with a higher risk of developing major depressive disorder before age 18. In youth, loss experiences have been linked to depressive symptoms, while events characterized by future threat were associated with anxiety [16]. Notably, there is a growing body of evidence suggesting the impact of LEs on mental health problems may be mediated by individuals’ cognitive appraisal of the event [17]. Thus, overall, there is evidence of an association between LEs exposure and mental health problems. Critically, however, underlying processes and mechanisms involved remain largely under-researched, especially in youth.

Elevated stress sensitivity in daily life has been proposed to be a transdiagnostic psychological mechanism contributing to the development and maintenance of mental health problems. Integrated models suggest that, as a consequence of exposure to socio-environmental risk (e.g., LEs), a process of gradual sensitization makes individuals more reactive to subsequent adversity as well as minor stressors in daily life (often referred to as elevated stress sensitivity or reactivity, which is partly related to other models, including the Diathesis-Stress Model or the Kindling hypothesis) [10, 18, 19]. It is thought that an increased sensitivity to minor stress in daily life may contribute to mental health problems and play a non-specific role in linking LEs and psychopathology in help-seeking individuals.

Context-sensitive ecological momentary assessment (EMA)—also known as experience sampling methodology—is a self-assessment diary technique that may be particularly well suited to test the proposed role of stress sensitivity on a behavioural level by investigating whether exposure to LEs is associated with heightened stress sensitivity [20]. In recent EMA studies, derived from the same sample as the present study, exposure to childhood trauma and bullying victimization [21, 22] was associated with elevated stress sensitivity in help-seeking youth. To our knowledge, however, no study has investigated the modifying effects of negative LEs on stress sensitivity in help-seeking youth. To investigate the role of stress sensitivity in linking exposure to LEs and mental disorders at a developmentally early stage of psychopathology, help-seeking adolescents and young adults (service users), their biological siblings, and control subjects were recruited.

The current study aimed to investigate whether exposure to negative LEs modifies stress sensitivity in youth’s daily life. Stress sensitivity was conceptualized as the associations between momentary stress (i.e., event-, activity-related and social stress combined) and (i) negative affect and (ii) psychotic experiences. We aimed to test the following primary hypotheses: first, we investigated whether overall lifetime as well as previous-year exposure to negative LEs (i.e., all exposure types combined) modifies individuals’ stress sensitivity within groups (service users, siblings, and control group) with greater associations when high vs*.* low exposure levels are compared. Second, we tested whether differences exist in the magnitude of associations when modifying effects of LEs exposure on stress sensitivity are compared across groups, with greater differences in service users vs. controls, service users vs. siblings, and siblings vs. controls. As secondary hypotheses, we examined whether lifetime and previous-year exposure to *specific types* of LEs (e.g., illness) modifies stress sensitivity within groups and whether there were differences of modifying effects across groups.

## Method

### Participants

Overall, 99 adolescents and young adults (age range 12–20 years) were recruited between April 2012 and March 2014, consisting of help-seeking service users, their biological siblings, and controls. The help-seeking individuals were recruited from secondary youth mental health services provided by the Mutsaers Foundation (MF) in Limburg, the Netherlands. Service users were included if they were currently receiving treatment from MF, and excluded if they had a DSM-IV autism spectrum disorder diagnosis (with the exception of pervasive developmental disorder not otherwise specified), IQ under 70, or insufficient command of Dutch. Moreover, biological siblings of participating service users were recruited. The same exclusion criteria applied, with the addition of lifetime history of receiving treatment from a mental health service. Lastly, individuals attending a school in the same catchment area as MF services were recruited as control subjects. Exclusion criteria were the same as for biological siblings. This study was granted ethical approval by the Ethical Review Committee of Maastricht University Medical Centre in Maastricht, the Netherlands (protocol number NL37420.068.11/METC11-3-060) and participants provided written informed consent.

### Measures

#### Socio-demographic characteristics

We collected data on age, sex, ethnicity, and education level using a socio-demographic schedule.

#### Life events

A modified version of the List of Threatening Experiences questionnaire (LTE) [23, 24] was used to assess negative LEs. In the current study, 19 differing LEs were grouped into 8 frequently used categories, which were based on the type of LEs exposure, including exposure to serious illness (e.g., *“hospitalisation or other medical treatments”*), experiences of loss (e.g., *“death of a family member or friend”*), and threatening events (e.g., *“involved in a serious accident in which you and/or someone else got hurt seriously”*). All items are provided in supplementary Table S1. In addition to asking whether individuals were exposed to LEs (dichotomous item), appraisal of LEs was also included. If an event has occurred (yes/no), participants were asked to rate the degree to which they perceived it as “unpleasant” or “pleasant” on a five-point scale (from 1 = *very unpleasant*; to 5 = *very pleasant*). As our focus was on negative LEs, events appraised as *neutral* or *pleasant* were coded as 0, while events appraised as *unpleasant* or *very unpleasant* were coded as 1 and 2, respectively. The recoded LTE items were used to calculate a total score that comprised our measure of overall exposure to the number of LEs (range 0–19), as well as continuous scores of appraisal ratings for specific types of LE (e.g., serious illness), to test primary and secondary hypotheses. In the current study, the LTE was modified to assess LEs from the previous 12 months as well as before age 17. Thus, lifetime prevalence of LEs was assessed in those under the age of 18 in addition to the previous year, but LEs prior to the age of 17 in those over the age of 18. The LTE has been found to have good psychometric properties [24]. In this study, Cronbach’s alpha was *α* = 0.62 for LEs in the previous 12 months and *α* = 0.76 for LEs before the age of 17, which is slightly lower than the internal consistency reported in other studies [24].

#### Depressive symptoms

The 21-item Beck Depression Inventory (BDI-II) [25] was used to assess depressive symptoms from the past 2-weeks on a 4-point scale (from 0 = *not present*; to 3 = *severe*). Good psychometric properties have been reported in clinical [26] and non-clinical [27] adolescent populations. In this study, Cronbach's alpha was *α* = 0.89.

#### Anxiety symptoms

A Dutch version [28] of the State-Trait Anxiety Inventory (STAI-Y) [29] was used to measure state and trait anxiety. The STAI-DY consists of two parts and demonstrates good reliability and moderate validity [30]. The first 20-item part (STAI-DY1) assesses state anxiety (current intensity; ranging from 1 = *not at all*; 4 = *very much*), whereas the second 40-item part (STAI-DY2) assesses trait anxiety (pervasive frequency; ranging from 1 = *rarely or never*; to 4 = *almost always*). In this study, Cronbach’s alpha was *α* = 0.89 for the STAI-DY1 and α = 0.94 for the STAI-DY2, respectively.

#### Psychotic symptoms

The 42-item Community Assessment of Psychic Experiences (CAPE) was used to assess the frequency (from 0 = *never*; to 3 = *nearly always*) and distress (from 0 = *not distressed*; to 3 = *very distressed*) of negative (14 items), positive (20 items), and depressive (8 items) dimensions of non-clinical, attenuated expressions of psychotic symptoms. CAPE has demonstrated good psychometric properties [31]. In this study, Cronbach's alpha was *α* = 0.93.

#### EMA measures

Daily changes in momentary stress (i.e., event-related, activity-related, and social stress), negative affect, and psychotic experiences were assessed using EMAs (range Cronbach's alpha for respective within-person centered variables, *α* = 0.65–0.79). This intensive self-assessment diary technique measures daily fine-grained subjective and social experiences with high ecological validity [20]. Data were collected outside the laboratory using a personal digital assistant, the “PsyMate”, which prompted participants with beeps ten times per day (between 7:30 am and 10:30 pm) at random intervals within set blocks of time of 90 min for 6 consecutive days. A detailed description of used EMA items is shown in Table 1. There has been evidence of high internal consistency and good concurrent validity for interviewer-rated measures of psychotic experiences and negative affect [32].

**Table 1:** EMA measures of stress, negative affect, and psychotic experiences

Domain	EMA measure
Momentary stress	Mean scores of event-related, activity-related, and social stress items were calculated and combined in form of a composite stress score to represent individuals’ momentary stress. Adequate concurrent validity with different stress measures has been reported [32]
Event	For event-related stress, participants had to rate the most important event since the last beep on a seven-point scale (from − 3 = *very unpleasant*; to + 3 = *very pleasant*). The item was reverse-coded such that higher ratings reflect higher levels of stress (− 3 coded as 7; + 3 coded as 1)
Activity	Activity-related stress was assessed by asking participants to identify what they were doing just before the beep (e.g., work or study, resting) and, subsequently, by asking whether they would “rather be doing something else”, whether “this activity is difficult” for them, and whether they believe they “can do this well” [reversed] on a seven-point scale (from 1 = *not at all*; to 7 = *very much*)
Social	Participants were asked about their current social context (e.g., “I am alone”, “I am with colleagues”, “I am with friends”). Social stress was assessed by asking participants to rate the items “I find the people I am with pleasant” [reversed; if with someone] or “I like to be alone” [reversed; if alone] on 7-point scale (from 1 = *not at all*; to 7 = *very much*)
Negative affect	Participants reported the degree to which they felt anxious, lonely, down, irritated, and insecure on a seven-point scale (from 1 = *not at all*; to 7 = *very much*). The mean of these five items constitute the negative affect score. Good psychometric properties have been reported for the EMA measure of negative affect [33]
Psychotic experiences	The mean scores of eight items about mental states related to psychotic experiences were used (seven-point scale from 1 = *not at all*; to 7 = *very much*). Participants were asked about the presence and intensity of hallucinations (e.g., “I hear things that aren’t really there”), thought problems (e.g., “My thoughts are influenced by others”, “It’s hard to express my thoughts in words”), delusional ideations and other states (e.g., “I feel suspicious/paranoid”, “I feel unreal”, “I feel harried”). The EMA measure for psychotic experiences has demonstrated good concurrent validity [33]

### Statistical analysis

In line with previous studies [21, 22], we first compared socio-demographic characteristics and standardized baseline scores (BDI-II, STAI-DY1/STAI-DY2, and CAPE) between groups (service users, biological siblings, and controls) using linear regression and *χ*^2^-tests. To account for statistical dependencies in EMA data resulting from the multilevel data structure (multiple observations nested within participants), linear mixed models were computed using the MIXED command in STATA 15. Momentary stress and group status were added as independent variables and (i) negative affect and (ii) psychotic experiences as outcome variables, while controlling for age, sex, ethnicity, and level of education. To test primary and secondary hypotheses, we added two-way (Stress*X*LE, Stress*X*Group, LE*X*Group) and three-way (Stress*X*LE*X*Group) interaction terms into the models. After the three-way interaction terms were added, increase in model fit was tested using Wald tests (TESTPARM command). To account for multiple testing, the *p* values of Wald tests were multiplied by the total number of tests to calculate family-wise error-corrected *p* values (*p*FWE) [33]. Next, we computed linear combinations of coefficients with the LINCOM command to test whether, within-groups, associations between momentary stress and (i) negative affect and (ii) psychotic experiences were greater in individuals exposed to high vs. low levels of exposure to LEs (by calculating standardized LEs scores: ± 1 *SD*, *M* = 0). Lastly, we explored whether effect moderation of LEs on stress sensitivity differs across groups by comparing differences in the magnitude of associations between momentary stress and (i) negative affect and (ii) psychotic experiences between individuals exposed to high vs. low levels of LEs exposure in service users vs. controls, service users vs*.* siblings, and siblings vs. controls, for all types of LE for which low prevalence does not preclude comparisons.

## Results

### Sample characteristics

In total, 109 individuals were eligible to participate. Of these, 99 youths (42 service users, 17 siblings, and 40 controls) completed the EMA with ≥ 20 valid responses over the 6-day assessment period as well as the LTE, BDI-II, CAPE, and STAI-DY1/DY2. Groups did not significantly differ on age, sex, ethnicity, or cannabis use, as well as the number of valid responses (Table 2). There was no evidence that the compliance changed over the course of the EMA assessments. However, there was evidence for higher levels of depression, state and trait anxiety, and negative and positive psychotic experiences, education, and attempted suicide in service users vs*.* controls and service users vs*.* siblings, respectively. As shown in Table 3, service users were exposed to higher overall levels of LEs during the last year compared to controls and siblings, while no differences were found comparing siblings and controls. A similar pattern of findings was demonstrated for overall LEs before age 17, with service users exposed to higher overall levels of LEs as compared to controls and siblings, while no significant differences were found comparing siblings and controls. This was also evident by looking at specific types of LEs during the last year and before the age of 17.

**Table 2:** Sample characteristics

	Service users (*n* = 42)	Siblings (*n* = 17)	Controls (*n* = 40)	Test statistic	*p*
Age (years), mean (SD)	15.4 (1.4)	15.3 (2.3)	15.6 (2.0)	*F* = 0.24, df = 2	0.785
Sex, *n* (%)					
Female	25 (59.5)	10 (58.8)	23 (57.5)	*χ*^2^ = 0.04, df = 2	0.983
Male	17 (40.5)	7 (41.2)	17 (42.5)		
Ethnicity, *n* (%)^a^					
White Dutch	26 (61.9)	11 (64.7)	25 (64.1)	*χ*^2^ = 0.06, df = 2	0.970
Other	16 (38.1)	6 (35.3)	14 (35.9)		
Level of education, *n* (%)^c^					
School	30 (71.4)	7 (41.2)	17 (42.5)	*χ*^2^ = 10.48, df = 2	0.033
Further	12 (28.6)	8 (47.1)	20 (50.0)		
Higher	–	2 (11.8)	3 (7.5)		
Cannabis use, *n* (%)					
12-months	9 (21.4)	1 (5.9)	4 (10.0)	*χ*^2^ = 3.36, df = 2	0.187
Lifetime	9 (21.4)	2 (11.8)	5 (12.5)	*χ*^2^ = 1.50, df = 2	0.473
Attempted suicide, *n* (%)					
During last year	6 (14.6)	–	–	–	–
Before age 17	8 (19.1)	–	–	–	–
DSM-IV diagnoses, *n* (%)					
Pervasive developmental disorders NOS	10 (23.8)	–	5 (12.5)	–	–
Attention-deficit and disruptive behaviour	6 (14.3)	3 (17.6)	–	–	–
Adjustment disorders	4 (9.5)	–	–	–	–
Anxiety disorders	2 (4.8)	–	–	–	–
Depressive disorders	2 (4.8)	–	–	–	–
Gender identity disorders	2 (4.8)	–	–	–	–
Learning disorders	–	–	2 (5.0)	–	–
Other disorders of infancy, childhood, or adolescence	5 (11.9)	–	–	–	–
Parent–child relational problem	5 (11.9)	1 (5.9)	1 (2.5)	–	–
Comorbid condition^b^	24 (57.1)	2 (11.8)	–	–	–
None	6 (14.3)	13 (76.5)	32 (80.0)	–	–
BDI-II sum sores, mean (SD)^a,d,e^	12.8 (9.2)	3.9 (3.3)	6.9 (7.0)	*F* = 10.5, df = 2	< 0.001
CAPE sum scores, mean (SD)^d,e^					
Positive	10.0 (9.4)	3.9 (3.2)	4.6 (3.9)	*F* = 8.28, df = 2	< 0.001
Negative	9.9 (6.7)	5.6 (3.8)	7.4 (4.8)	F = 3.88, df = 2	0.024
Depressive	7.7 (4.0)	4.2 (1.8)	4.7 (3.4)	*F* = 9.90, df = 2	< 0.001
STAI-DY1 (state anxiety)^a^ sum scores, mean (SD)^d,e^	35.5 (10.6)	30.2 (6.8)	31.1 (7.2)	*F* = 3.47, df = 2	0.035
STAI-DY2 (trait anxiety)^a^ sum scores, mean (SD)^d,e^	85.6 (20.8)	67.1 (9.2)	74.1 (16.4)	*F* = 8.12, df = 2	< 0.001
Number of valid beeps, mean (range, min–max)	43.8 (24–59)	42.8 (23–57)	44.9 (25–58)	*F* = 0.23, df = 2	0.764

	Service users vs. controls		Siblings vs. controls		Service users vs. siblings	
	*β* (95% CI)	*p*	*β* (95% CI)	*p*	*β* (95% CI)	*p*
BDI-II	0.71 (0.30–1.11)	0.001	− 0.37 (− 0.89–0.16)	0.168	1.08 (0.55–1.60)	< 0.001
CAPE						
Positive	0.75 (0.34–1.17)	< 0.001	− 0.09 (− 0.63–0.44)	0.735	0.84 (0.31–1.38)	0.002
Negative	0.42 (− 0.01–0.85)	0.057	− 0.31 (− 0.87–0.25)	0.269	0.73 (0.17–1.29)	0.011
Depressive	0.80 (0.40–1.21)	< 0.001	− 0.12 (− 0.64–0.41)	0.666	0.92 (0.39–1.45)	0.001
STAI-DY1	0.50 (0.06–0.93)	0.025	− 0.09 (− 0.65–0.47)	0.748	0.59 (0.03–1.14)	0.040
STAI-DY2	0.59 (0.19–0.99)	0.004	− 0.36 (− 0.88–0.16)	0.170	0.95 (0.43–1.46)	< 0.001

*SD* standard deviation, *df* degrees of freedom, *β* standardized regression coefficients (mean score differences), *vs.* versus, *CI* confidence interval

^a^Missing values: ethnicity = 1, BDI = 1, STAI-DY1 = 1, STAI-DY2 = 2

^b^Consisting of the following diagnostic categories in the service users group: Additional codes (Parent–child relational problem, 33.3%; Borderline intellectual functioning, 13.3%; Neglect of child, 6.7%), Attention-deficit and disruptive behaviour disorders (10%), Learning disorders (10%), Personality disorders (6.7%), Mild mental retardation (6.7%), Anxiety disorders (3.3%), Dissociative disorders (3.3%), Tic disorders (3.3%), Amphetamine related disorders (3.3%)

^c^Categories defined as: school (primary education, LBO, MAVO, VMBO), further (MBO, HAVO, VWO), and higher (HBO, WO) education of the Dutch educational system

^d^Cut-off scores of clinically significant severity: BDI-II = total score above 13; STAI-DY1 = score above 40

^e^Standardized mean score differences across groups

**Table 3:** Exposure to life events during the last year as well as before the age of 17 within and across groups

	Service users (*n* = 42)	Siblings (*n* = 17)	Controls (*n* = 40)	Service users vs*.* controls	Service users vs*.* siblings	Siblings vs*.* controls
Exposure to life events during the last year, mean (SD; range)						
Any	3.45 (3.67; 0–19)	1.82 (2.21; 0–9)	1.52 (1.96; 0–9)	*β* = 0.64 (0.22–1.06)*	*β* = 0.54 (– 0.001–1.09)*	*β* = 0.10 (− 0.45–0.65)
Illness	1.00 (1.49; 0–5)	0.52 (0.87;0–2)	0.65 (0.83;0–2)	*β* = 0.29 (− 0.13–0.73)	*β* = 0.40 (– 0.16–0.96)	*β* = − 0.10 (– 0.67–0.46)
Loss	0.48 (0.89; 0–3)	0.47 (0.87; 0–2)	0.52 (0.90; 0–3)	*β* = − 0.05 (– 0.49 – 0.38)	*β* = 0.006 (– 0.56–0.58)	*β* = − 0.06 (− 0.64–0.51)
Conflict	0.95 (1.48; 0–6)	0.47 (0.87; 0–2)	0.2 (0.51; 0–2)	*β* = 0.66 (0.24–1.08)*	*β* = 0.42 (– 0.12–0.97)	*β* = 0.24 (− 0.31–0.79)
Occupation	0.33 (0.72; 0–2)	–	0.05 (0.22; 0–1)	*β* = 0.55 (0.13–0.98)*	*β* = 0.65 (0.10–1.2)*	*β* = − 0.09 (− 0.65–0.45)
Finance	0.09 (0.43; 0–2)	–	–	*β* = 0.22 (– 1.0–0.77)	*β* = 0.33 (– 0.23–0.90)	*β* = -0.00 (− 0.57–0.57)
Housing	–	–	–			
Legal	0.14 (0.52; 0–2)	0.05 (0.24; 0–1)	–	*β *= 0.39 (− 0.03–0.83)	*β* = 0.23 (− 0.33–0.80)	*β* = 0.16 (− 0.40–0.73)
Threat	0.45 (0.94; 0–4)	0.29 (0.98; 0–4)	0.1 (0.44; 0–2)	*β* = 0.44 (0.008–0.87)*	*β* = 0.19 (− 0.36–0.76)	*β* = 0.24 (− 0.32–0.81)
Exposure to life events before the age of 17, mean (SD; range)						
Any	6.35 (3.27; 0–15)	4.29 (2.08; 0–8)	3.47 (2.47; 0–8)	*β* = 0.93 (0.54–1.33)**	*β* = 0.67 (0.15–1.19)*	*β* = 0.26 (− 0.25–0.78)
Illness	1.64 (1.35)	1.47 (0.94)	1.00 (1.06)	*β* = 0.53 (0.10–0.96)*	*β* = 0.14 (− 0.41–0.70)	*β* = 0.39 (− 0.17–0.95)
Loss	1.28 (1.21; 0–4)	1.64 (1.45; 0–4)	1.17 (1.23; 0–4)	*β* = 0.08 (− 0.35–0.52)	*β* = − 0.28 (− 0.85–0.28)	*β* = 0.37 (− 0.20–0.94)
Conflict	1.64 (1.72; 0–9)	0.82 (1.07; 0–3)	0.62 (1.14; 0–6)	*β* = 0.68 (0.26–1.10)*	*β* = 0.55 (0.009–1.09)*	*β* = 0.13 (− 0.41–0.68)
Occupation	0.45 (0.80; 0–2)	–	0.12 (0.46; 0–2)	*β* = 0.52 (0.10–0.94)*	*β* = 0.72 (0.17–1.27)*	*β* = − 0.20 (− 0.75–0.35)
Finance	0.19 (0.59; 0–2)	–	0.02 (0.15; 0–1)	*β* = 0.40 (− 0.02–0.84)	*β* = 0.46 (− 0.09–1.03)	*β* = − 0.06 (− 0.62–0.50)
Housing	–	–	–			
Legal	0.21 (0.52; 0–2)	–	–	*β* = 0.60 (0.18–1.03)*	*β* = 0.60 (0.05–1.15)*	*β* = − 0.00 (− 0.55–0.55)
Threat	0.92 (1.23; 0–4)	0.35 (0.79; 0–2)	0.52 (0.85; 0–3)	*β* = 0.38 (− 0.04–0.81)	*β* = 0.55 (− 0.008–1.11)	*β* = − 0.16 (− 0.73–0.40)

*SD* standard deviation, *β* standardized regression coefficients (mean score differences), *vs.* versus, *CI* confidence interval

*p < 0.05; **p < 0.001

### Association between momentary stress and negative affect by LEs exposure and group

There was evidence in support of primary hypotheses that overall lifetime as well as previous-year exposure to LEs modified the association between momentary stress and negative affect (Table 4), as indicated by significant three-way interaction effects described below.

**Table 4:** Association of momentary stress with negative affect and psychotic experiences, by levels of exposure to life events in service users, siblings, and controls^a^

	Service users		Siblings		Controls		Wald test for interaction^c^		
	adj. *β* (95% CI)	*p*	adj. *β* (95% CI)	*p*	adj. *β* (95% CI)	*p*	*χ*^2^ (df)	*p*	*p*FWE
*Outcome: negative affect*									
Momentary stress^b^ × life events × group									
Overall exposure to life events during the last year							15.12 (2)	< 0.001	0.004
High (mean + 1 SD)	0.28 (0.24–0.31)	< 0.001	0.08 (− 0.02–0.19)	0.130	0.16 (0.09–0.24)	< 0.001			
Average (mean)	0.19 (0.15–0.22)	< 0.001	0.08 (0.02–0.14)	0.010	0.18 (0.14–0.22)	< 0.001			
Low (mean-1 SD)	0.10 (0.05–0.15)	< 0.001	0.07 (− 0.03–0.17)	0.143	0.20 (0.14–0.25)	< 0.001			
High vs. low^d^	0.18 (0.13–0.22)	< 0.001	0.01 (− 0.16–0.18)	0.903	− 0.03 (− 0.14–0.07)	0.539			
Overall exposure to life events before age 17							7.53 (2)	0.023	n.s.
High (mean + 1 SD)	0.28 (0.24–0.32)	< 0.001	0.09 (− 0.02–0.20)	0.116	0.19 (0.11–0.26)	< 0.001			
Average (mean)	0.20 (0.16–0.24)	< 0.001	0.08 (0.02–0.14)	0.011	0.18 (0.14–0.23)	< 0.001			
Low (mean-1 SD)	0.11 (0.05–0.18)	< 0.001	0.07 (− 0.03–0.17)	0.164	0.18 (0.13–0.23)	< 0.001			
High vs. low^d^	0.16 (0.09–0.23)	< 0.001	0.02 (− 0.15–0.19)	0.851	0.01 (− 0.10–0.10)	0.916			
*Outcome: psychotic experiences*									
Momentary stress^b^ × life events × group									
Overall exposure to life events during the last year							11.75 (2)	0.003	0.012
High (mean + 1 SD)	0.14 (0.12–0.16)	< 0.001	0.01 (− 0.05–0.07)	0.668	0.05 (0.01–0.10)	0.009			
Average (mean)	0.09 (0.07–0.11)	< 0.001	0.03 (− 0.00–0.06)	0.073	0.05 (0.03–0.07)	< 0.001			
Low (mean-1 SD)	0.05 (0.02–0.08)	< 0.001	0.05 (− 0.01–0.10)	0.085	0.05 (0.02–0.08)	0.002			
High vs*.* low^d^	0.09 (0.06–0.11)	< 0.001	− 0.03 (− 0.13–0.06)	0.466	0.01 (− 0.05–0.06)	0.837			
Overall exposure to life events before age 17							8.36 (2)	0.015	n.s.
High (mean + 1 SD)	0.14 (0.12–0.16)	< 0.001	0.03 (− 0.03–0.08)	0.395	0.06 (0.02–0.10)	0.002			
Average (mean)	0.09 (0.07–0.12)	< 0.001	0.03 (− 0.00–0.06)	0.066	0.05 (0.03–0.08)	< 0.001			
Low (mean-1 SD)	0.04 (0.01–0.08)	0.011	0.04 (− 0.02–0.09)	0.187	0.04 (0.01–0.07)	0.002			
High vs. low^d^	0.10 (0.06–0.14)	< 0.001	− 0.01 (− 0.10–0.08)	0.815	0.02 (− 0.03–0.07)	0.456			

	Service users vs. controls^d^		Siblings vs. controls^d^		Service users vs. siblings^d^	
	adj. *β* (95% CI)	*p*	adj. *β* (95% CI)	*p*	adj. *β* (95% CI)	*p*
Δ high vs. low exposure levels of life events across groups						
*Outcome: negative affect*						
Momentary stress × life events × group						
Overall exposure to life events during the last year	0.21 (0.10–0.32)	< 0.001	0.17 (− 0.02–0.36)	0.083	0.17 (− 0.01–0.34)	0.064
Overall exposure to life events before the age of 17	0.16 (0.04–0.28)	0.011	0.01 (− 0.18–0.21)	0.912	0.15 (− 0.04–0.33)	0.118
*Outcome: psychotic experiences*						
Momentary stress × life events × group						
Overall exposure to life events during the last year	0.08 (0.02–0.14)	0.010	− 0.04 (− 0.15–0.07)	0.466	0.12 (0.03–0.22)	0.012
Overall exposure to life events before the age of 17	0.08 (0.01–0.14)	0.018	− 0.03 (− 0.14–0.07)	0.571	0.11 (0.01–0.21)	0.031

*SD* standard deviation, *df* degrees of freedom, *vs.* versus, *n.s.* non-significant, *CI* confidence interval, *adj. β*: standardized regression coefficients, continuous independent variables were standardized (mean = 0, SD = 1) for interpreting significant three-way interaction terms and examining the difference in associations between high (mean + 1 SD), average (mean), and low (mean – 1 SD) levels of exposure to life events within and across groups (service users, siblings, controls), *pFWE* family-wise error-corrected *p* values were computed by multiplying the unadjusted *p* value by the total number of tests (primary hypotheses: *N* = 4) to adjust significance levels of likelihood ratio tests for three-way interactions

^a^Adjusted for age, sex, ethnicity, and level of education

^b^Momentary stress was calculated by combining the ratings of items assessing event-related, activity-related, and social stress

^c^Three-way interaction as included in the following model (with y_ij_ for negative affect or psychotic experiences as outcome variable): *y*_*ij*_ = *β*_0_ + *β*_1_(STRESS_*ij*_) + *β*_2_(LIFE EVENTS_*j*_) + *β*_3_(GROUP_*j*_) + *β*_4_(STRESS_*ij*_ × LIFE EVENTS_*j*_) + *β*_5_(STRESS_*ij*_ × GROUP_*j*_) + *β*_6_(LIFE EVENTS_*j*_ × GROUP_*j*_) + *β*_7_(STRESS_*ij*_ × LIFE EVENTS_*j*_ × GROUP_*j*_) + *ε*_*ij*_ (full model not shown—available upon request)

^d^Difference in the magnitude of associations of momentary stress with psychotic experiences between those exposed to high vs. low levels of exposure to life events across groups (Δ high vs. low)

#### Within-group comparisons

Momentary stress was associated with increased negative affect in service users (*adj. β* = 0.18, *p* < 0.001) when high vs*.* low levels of exposure to overall LEs during the last year were compared, while no significant differences by exposure levels were found in siblings and controls. When comparing high vs*.* low levels of exposure to overall LEs before age 17, momentary stress was associated with increased negative affect in service users (*adj. β* = 0.16, *p* < 0.001), while no significant differences were found in siblings and controls. Analyses to test secondary hypotheses (Table 5) revealed that momentary stress was associated with lower negative affect in the control group when comparing high vs*.* low levels of exposure to threatening events (*adj. β* = − 0.14, *p* = 0.012). In contrast, increased negative affect in response to stress was observed in service users comparing high vs*.* low levels of exposure to loss experiences (*adj. β* = 0.21, *p* < 0.001), conflict events (*adj. β* = 0.17, *p* < 0.001), and threatening events (*adj. β* = 0.09, *p* < 0.001), while no significant differences were demonstrated in siblings.

**Table 5:** Association of momentary stress with negative affect and psychotic experiences, by levels of exposure to life events in service users, siblings, and controls^a^

	Service users		Siblings			Controls				Wald test for interaction^c^					
	adj. *β* (95% CI)	*p*	adj. *β* (95% CI)		*p*	adj. *β* (95% CI)		*p*		*χ*^2^ (df)		*p*		*p*FWE	
*Outcome: negative affect*															
Momentary stress^b^ × life events × group															
Exposure during the last year:															
Illness										0.71 (2)		n.s.		n.s.	
Loss										24.59 (2)		< 0.001		0.007	
High (mean + 1 SD)	0.34 (0.29–0.39)	< 0.001	0.11 (0.04–0.18)		0.003	0.16 (0.10–0.21)		< 0.001							
Average (mean)	0.23 (0.20–0.27)	< 0.001	0.07 (0.01–0.13)		0.029	0.18 (0.14–0.22)		< 0.001							
Low (mean-1 SD)	0.13 (0.08–0.18)	< 0.001	0.03 (− 0.06–0.12)		0.532	0.21 (0.15–0.26)		< 0.001							
High vs. low^d^	0.21 (0.13–0.28)	< 0.001	0.08 (− 0.03–0.19)		0.149	− 0.05 (− 0.12–0.03)		0.195							
Conflict										13.18 (2)		0.002		0.014	
High (mean + 1 SD)	0.27 (0.23–0.31)	< 0.001	0.04 (− 0.06–0.14)		0.474	0.18 (0.07–0.28)		0.001							
Average (mean)	0.18 (0.15–0.22)	< 0.001	0.08 (0.02–0.14)		0.012	0.18 (0.14–0.23)		< 0.001							
Low (mean-1 SD)	0.10 (0.04–0.15)	< 0.001	0.12 (0.02–0.22)		0.017	0.19 (0.12–0.26)		< 0.001							
High vs. low^d^	0.17 (0.13–0.22)	< 0.001	− 0.08 (− 0.24–0.08)		0.308	− 0.01 (− 0.16–0.14)		0.902							
Occupation										0.30 (1)		n.s.		n.s.	
Finance	–		–			–				–		–		–	
Housing	–		–			–				–		–		–	
Legal problems	–		–			–				–		–		–	
Threat										15.38 (2)		< 0.001		0.007	
High (mean + 1 SD)	0.26 (0.22–0.30)	< 0.001	0.07 (− 0.01–0.15)		0.083	0.11 (0.03–0.18)		0.005							
Average (mean)	0.22 (0.18–0.25)	< 0.001	0.08 (0.02–0.14)		0.010	0.18 (0.14–0.22)		< 0.001							
Low (mean-1 SD)	0.17 (0.12–0.22)	< 0.001	0.09 (0.01–0.17)		0.030	0.25 (0.18–0.31)		< 0.001							
High vs. low^d^	0.09 (0.04–0.14)	0.001	− 0.02 (− 0.12–0.09)		0.745	− 0.14 (− 0.25–− 0.03)		0.012							
Exposure before age 17:															
Illness										2.48 (2)		n.s.		n.s.	
Loss										0.27 (2)		n.s.		n.s.	
Conflict										2.77 (2)		n.s.		n.s.	
Occupation										0.39 (1)		n.s.		n.s.	
Finance										0.40 (1)		n.s.		n.s.	
Legal	–					–				–		–		–	
Threat										16.14 (2)		< 0.001		0.005	
High (mean + 1 SD)	0.29 (0.25–0.33)	< 0.001	0.01 (− 0.09–0.11)		0.800	0.16 (0.09–0.24)		< 0.001							
Average (mean)	0.22 (0.18–0.25)	< 0.001	0.07 (0.01–0.13)		0.024	0.18 (0.14–0.22)		< 0.001							
Low (mean-1 SD)	0.15 (0.09–0.20)	< 0.001	0.13 (0.04–0.22)		0.004	0.20 (0.14–0.25)		< 0.001							
High vs. low^d^	0.14 (0.08–0.20)	< 0.001	− 0.12 (− 0.26–0.03)		0.112	− 0.03 (− 0.14–0.07)		0.517							
*Outcome: psychotic experiences*															
Momentary stress^b^ × life events × group															
Exposure during the last year:															
Illness										5.47 (2)		n.s.		n.s.	
Loss										16.13 (2)		< 0.001		0.005	
High (mean + 1 SD)	0.17 (0.14–0.19)	< 0.001	0.03 (− 0.01–0.07)		0.136	0.05 (0.02–0.08)		0.001							
Average (mean)	0.12 (0.10–0.14)	< 0.001	0.03 (− 0.00–0.07)		0.064	0.05 (0.03–0.07)		< 0.001							
Low (mean-1 SD)	0.07 (0.04–0.09)	< 0.001	0.03 (− 0.02–0.08)		0.183	0.05 (0.02–0.08)		0.001							
High vs. low^d^	0.10 (0.06–0.14)	< 0.001	− 0.00 (− 0.06–0.06)		0.908	− 0.00 (− 0.04–0.04)		0.969							
Conflict										2.50 (2)		n.s.		n.s.	
Occupation										0.26 (2)		n.s.		n.s.	
Finance	–		–			–				–		–		–	
Housing	–		–			–				–		–		–	
Legal										–		–		–	
Threat										8.97 (2)		0.011		n.s.	
High (mean + 1 SD)	0.14 (0.12–0.16)	< 0.001	0.02 (− 0.02–0.06)		0.408	0.06 (0.03–0.10)		0.001							
Average (mean)	0.10 (0.09–0.12)	< 0.001	0.03 (− 0.00–0.06)		0.062	0.05 (0.03–0.07)		< 0.001							
Low (mean-1 SD)	0.07 (0.04–0.10)	< 0.001	0.04 (0.00–0.09)		0.043	0.04 (0.01–0.07)		0.021							
High vs. low^d^	0.07 (0.04–0.09)	< 0.001	− 0.03 (− 0.08–0.03)		0.361	0.02 (− 0.03–0.08)		0.411							
Exposure before age 17:															
Illness										0.12 (2)		n.s.		n.s.	
Loss										3.63 (2)		n.s.		n.s.	
Conflict										5.13 (2)		n.s.		n.s.	
Occupation											0.04 (1)		n.s.		n.s.
Finance											0.14 (1)		n.s.		n.s.
Legal	–			–			–				–		–		–
Threat											5.66 (2)		0.059		n.s.
High (mean + 1 SD)	0.14 (0.12–0.16)	< 0.001		0.03 (− 0.03–0.08)	0.350		0.05 (0.01–0.09)		0.012						
Average (mean)	0.11 (0.09–0.13)	< 0.001		0.03 (− 0.02–0.06)	0.072		0.05 (0.03–0.07)		< 0.001						
Low (mean-1 SD)	0.08 (0.05–0.11)	< 0.001		0.04 (− 0.01–0.08)	0.142		0.05 (0.02–0.08)		0.002						
High vs. low^d^	0.06 (0.03–0.10)	< 0.001		− 0.01 (− 0.09–0.07)	0.861		0.00 (− 0.06–0.06)		0.977						

	Service users vs. controls^d^			Siblings vs. controls^d^			Service users vs. siblings^d^	
	adj. *β* (95% CI)	*p*		adj. *β* (95% CI)	*p*		adj. *β* (95% CI)	*p*
Δ high vs low exposure levels of life events across groups								
*Outcome: negative affect*								
Momentary stress × life events × group								
During the last year:								
Loss	0.26 (0.15–0.36)	< 0.001		0.13 (− 0.00–0.26)	0.055		0.13 (− 0.00–0.26)	0.054
Conflict	0.18 (0.02–0.35)	0.026		− 0.07 (− 0.29–0.15)	0.523		0.26 (0.09 – 0.42)	0.002
Threat	0.23 (0.11–0.35)	< 0.001		0.12 (− 0.03–0.27)	0.108		0.11 (− 0.01–0.22)	0.066
Before age 17:								
Threat	0.18 (0.06–0.30)	0.004		− 0.08 (− 0.26–0.10)	0.367		0.26 (0.10–0.41)	0.001
*Outcome: psychotic experiences*								
Momentary stress × life events × group								
During the last year:								
Loss	0.10 (0.05–0.16)	< 0.001		− 0.00 (− 0.07–0.07)	0.941		0.10 (0.03–0.17)	0.003
Threat	0.04 (− 0.02–0.11)	0.211		− 0.05 (− 0.13–0.03)	0.220		0.09 (0.03–0.15)	0.004
Before age 17:								
Threat	0.06 (− 0.00–0.13)	0.053		− 0.01 (− 0.11–0.09)	0.838		0.07 (− 0.01–0.16)	0.082

*SD* standard deviation, *df* degrees of freedom, *vs.* versus, *n.s.* non-significant, *CI* confidence interval, *adj. β* standardized regression coefficients, continuous independent variables were standardized (mean = 0, SD = 1) for interpreting significant three-way interaction terms and examining the difference in associations between high (mean + 1 SD), average (mean), and low (mean – 1 SD) levels of exposure to life events within and across groups (service users, siblings, controls), *pFWE* family-wise error-corrected *p* values were computed by multiplying the unadjusted *p* value by the total number of tests (secondary hypotheses: *N* = 7) to adjust significance levels of likelihood ratio tests for three-way interactions

^a^Adjusted for age, se, ethnicity, and level of education

^b^Momentary stress was calculated by combining the ratings of items assessing event-related, activity-related, and social stress

^c^Three-way interaction as included in the following model (with *y*_*ij*_ for negative affect or psychotic experiences as outcome variable): *y*_*ij*_ = *β*_0_ + *β*_1_(STRESS_*ij*_) + *β*_2_(LIFE EVENTS_*j*_) + *β*_3_(GROUP_*j*_) + *β*_4_(STRESS_*ij*_ × LIFE EVENTS_*j*_) + *β*_5_(STRESS_*ij*_ × GROUP_*j*_) + *β*_6_(LIFE EVENTS_*j*_ × GROUP_*j*_) + *β*_7_(STRESS_*ij*_ × LIFE EVENTS_*j*_ × GROUP_*j*_) + *ε*_*ij*_ (full model not shown—available upon request)

^d^Difference in the magnitude of associations of momentary stress with psychotic experiences between those exposed to high vs. low levels of exposure to life events across groups (Δ high vs. low)

#### Between-group comparisons

To investigate whether the modifying effects of exposure to LEs on stress sensitivity differed between groups, differences in magnitude of associations between those exposed to high vs*.* low levels of LEs were examined across groups. Specifically, the difference in magnitude of associations between stress and negative affect was greater in service users than in controls when comparing high vs*.* low levels of exposure to overall LEs during the last year (*adj. β* = 0.21, *p* < 0.001) and before age 17 (*adj. β* = 0.16, *p* = 0.011). Furthermore, in testing secondary hypotheses (Table 5), we found significant differences in the magnitude of associations between those exposed to high vs*.* low levels of experience of loss (*adj. β* = 0.26, *p* < 0.001), conflict events (*adj. β* = 0.18, *p* = 0.026), and threatening events (*adj. β* = 0.23, *p* < 0.001) during the last year comparing service users vs*.* controls. Moreover, the difference in magnitude of associations between stress and negative affect was greater in service users than siblings comparing high vs*.* low exposure to conflict events during the last year (*adj. β* = 0.26, *p* = 0.002), and threatening events (*adj. β* = 0.26, *p* = 0.001) and, at trend level, experiences of loss (*adj. β* = 0.13, *p* = 0.054) before age 17. No significant differences were found comparing siblings vs*.* controls.

### Association between stress and psychotic experiences by LEs exposure and group

There was evidence in support of the primary hypotheses that exposure to overall LEs modified the association between momentary stress and psychotic experiences (Table 4), as indicated by significant three-way interaction effects described below.

#### Within-group comparisons

Momentary stress was associated with more intense psychotic experiences in service users (*adj. β* = 0.09, *p* < 0.001) exposed to high levels of overall LEs compared to those with low exposure levels during the last year, while no differences were found in siblings and controls. When considering overall exposure to LEs before age 17, momentary stress was associated with more intense psychotic experiences in service users (*adj. β* = 0.10, *p* < 0.001) when high vs*.* low overall LEs levels were compared, while no significant differences were found in siblings and controls. Analyses of secondary hypotheses (Table 5) revealed that stress was significantly associated with more intense psychotic experiences during the last year when comparing service users exposed to high vs*.* low levels of experiences of loss (*adj. β* = 0.10, *p* < 0.001) and threatening events (*adj. β* = 0.07, *p* < 0.001). A similar pattern of findings was evident in service users with high vs*.* low exposure to threatening events (*adj. β* = 0.06, *p* < 0.001) before age 17. In contrast, stress was not significantly associated with more intense psychotic experiences across any type or level of LEs in neither the control group nor the sibling group (Table 5).

#### Between-group comparisons

There were differences in the magnitude of associations between momentary stress and psychotic experiences in those exposed to high vs*.* low levels of overall LEs comparing service users vs*.* controls during the last year (*adj. β* = 0.08, *p* = 0.010) and before age 17 (*adj. β* = 0.08, *p* = 0.018). Moreover, the difference in magnitude of associations between momentary stress and psychotic experiences was greater in service users than in siblings when high vs*.* low levels of exposure to overall LEs during the last year (*adj. β* = 0.12, *p* = 0.012) as well as before age 17 (*adj. β* = 0.11, *p* = 0.031) were compared (Table 4). However, there was no difference when high vs*.* low levels of exposure to overall LEs were compared in siblings vs*.* controls. Analyses of secondary hypotheses (Table 5) showed differences in magnitude of associations between momentary stress and psychotic experiences comparing high vs*.* low exposure levels to experiences of loss (*adj. β* = 0.10, *p* < 0.001), but not threatening events (*adj. β* = 0.04, *p* = 0.211), during the last year in service users vs*.* controls. However, service users marginally differed from controls in the magnitude of associations between momentary stress and psychotic experiences by exposure levels to threatening events before age 17 (*adj. β* = 0.06, *p* = 0.053). Lastly, service users differed significantly from siblings in magnitude of associations between momentary stress and psychotic experiences comparing high vs*.* low levels of exposure to experiences of loss (*adj. β* = 0.10, *p* = 0.003) and threatening events *(adj. β* = 0.09, *p* = 0.004) during the last year. No significant differences were found comparing siblings vs*.* controls.

## Discussion

### Main findings

There was evidence that lifetime and previous-year exposure to overall as well as specific types of LEs modified stress sensitivity in help-seeking service users. Service users experienced increased negative affect and more intense psychotic experiences in response to minor daily stress when high vs*.* low exposure levels were compared. In siblings, however, we found no evidence that overall exposure to as well as specific types of LEs modified stress sensitivity. In controls, secondary analyses revealed decreased negative affect in response to stress comparing those exposed to high vs*.* low levels of threatening experiences. Thus, our findings tentatively suggest that individuals’ response to minor stress in daily life may represent a putative risk mechanism through which exposure to LEs influence youth mental health.

### Methodological considerations

The reported findings should be interpreted in view of potential limitations. First, the LTE is a retrospective measure of self-reported LEs. Consequently, recall bias and cognitive distortion may have influenced reported findings [34]. Additionally, current mental health problems may have influenced how LEs were appraised, and thus on the interpretation of reported findings, particularly in service users. However, there is evidence that service users report adverse childhood experiences similarly to controls and that self-report questionnaires used in retrospective studies have comparable validity [35]. To account for the effect of symptom severity on reporting exposure to LEs, longitudinal data would be required, which would necessitate a larger sample size and a cohort design. Furthermore, it may have been difficult for participants to differentiate LEs from within the last 12 months from LEs before age 17. However, the low sample age likely minimized the impact of biases on reported LEs, as the time between exposure to and time of assessments of LEs was limited. Similarly, EMAs were based on self-report. While this allows for ecologically valid measurements of momentary stress, context, and experiences on the behavioural level, future research should consider triangulating the impact of LEs on individuals’ stress sensitivity across other levels of investigation, such as biological markers [36] and passively collected digital sensor data [37]. Secondly, some of the LEs included in the current study (e.g., housing, financial, and legal problems) were only prevalent in few or none of participating individuals. Consequently, we were not able to test modifying effects of all types of LEs on stress sensitivity in secondary analyses. Third, assessment burden associated with EMAs may have introduced selection bias. However, studies have demonstrated that EMAs are reliable and feasible in adult as well as adolescent clinical and community populations [20]. Additionally, extensive briefing on the “PsyMate” and EMA procedure ensured a high number of valid responses (90.8%). Fourth, the group of siblings was relatively small (*n* = 17) and findings that LEs did not modify stress sensitivity in this intermediate risk group may have occurred because of sampling error. Fifth, despite adjustment for potential confounders (i.e., age, sex, education level, and ethnicity), other unmeasured factors such as socioeconomic status, personality traits, and polygenetic risk for psychopathologies, to name a few, could have influenced reported findings [38]. Furthermore, analyses were not adjusted for shared genetic and socio-environmental risk factors among service users and siblings, as data used to test primary and secondary hypotheses did not include any shared genetic markers or records of shared LEs. Future studies may further investigate the impact of clustering of LEs within families on individuals’ stress sensitivity. Sixth, some of the reported findings (e.g., effect modification of experiences of loss on service users’ stress sensitivity) were below conventional alpha. The null hypothesis significance testing paradigm—and the *p* value threshold intrinsic to it—is currently strongly debated with widely differing views [39–41]. As a result, reported findings should be interpreted as suggestive rather than conclusive evidence. Large EMA studies with sufficient power are required to replicate reported findings. Seventh, the data collected over 6 days with EMA was modelled cross-sectionally and temporality of stress, negative affect, and psychotic experiences was not specifically investigated [42]. It is recommended that future studies consider time-lagged and moderated mediation models to further investigate temporality as one important criterion important to inferring causality. More research is needed to examine the timing, mechanism, and outcome of exposure to LEs through well-controlled cohort studies to determine whether increased stress sensitivity mediates the association between exposure and the onset of mental disorders. The potential buffering effect of protective factors (e.g., number of close relationships, coping skills, and personality traits) on stress sensitivity, as well as complex interactions with other socio-environmental and genetic risk factors, may be tested using this study design. Lastly, the internal consistency of the LTE measure found in our study appeared to be lower when compared to previous studies [24]. More studies are needed to investigate the reliability of the LTE in younger age groups.

### Comparison with previous research

Previous research suggests exposure to adverse childhood experiences, including LEs [6–9, 11–14, 16, 43], is associated with an increased risk of developing mental health problems. However, candidate mechanisms remain poorly understood, particularly in youth. By contributing to lasting changes to the way an individual responds to stress, *stress sensitization* has been proposed to be a common mechanistic pathway that may help explain the relation between exposure to socio-environmental risk and psychopathology [18]. Notably, this proposition has already been investigated in adult populations. Adults with mental disorders (e.g., depression, psychosis) and a history of childhood trauma and LEs were found to have elevated affective and psychotic reactivity in response to minor daily stressors [32, 44]. Thus, stress sensitization may represent a putative mechanism underlying exposure to LEs and psychopathology. However, whether these findings can be generalized to young help-seeking individuals remained largely under-researched.

The current study is the first to report that young help-seeking individuals who were exposed to high levels of LEs responded to minor stressors in daily life with increased negative affect and psychotic experiences as compared to those with low exposure levels. Interestingly, these findings are in line with reported effects of childhood trauma [21] and bullying victimization [22] on stress sensitivity derived from the same sample, and with findings including individuals with an at-risk mental state (ARMS) for psychosis and first-episode psychosis exposed to childhood trauma [32]. Thus, in line with previous research, the present study suggests, although not directly tested, that behavioural sensitization in form of an increased stress sensitivity in daily life may be associated with exposure to ACEs and may have downstream contributions along multiple pathways leading to poor mental health.

In contrast, exposure to LEs before age 17 did not modify individuals’ stress sensitivity in control subjects. However, in secondary analyses, controls exposed to high levels of more intrusive LEs (i.e., threatening experiences, serious accident) during the previous year responded with *decreased* negative affect as compared to those with low exposure to LEs. In other words, controls appeared to be more resilient to the detrimental effects of high exposure to more recent threatening LEs when compared to low exposure levels. This is interesting as it mirrors previous findings in which physical abuse and neglect [21], physical bullying [22], and sexual abuse [32] were associated with decreased negative affect in response to stress in controls. Taken together, high levels of exposure to more intrusive ACEs may result in resilience against subsequent minor stressors in daily life in individuals who do not develop help-seeking behaviour.

In biological siblings of service users, we found no evidence that exposure to LEs modifies stress sensitivity. Specifically, when comparing high vs. low exposure to LEs, siblings did not respond to minor stressors in daily life with an increased negative affect and psychotic experiences. However, with a small sample size, we have to be cautious when interpreting these findings. Moreover, findings at the group-level are possibly influenced by mixed resiliency to stress sensitivity at the person-level considering that siblings form an *intermediate* risk group and have a higher liability to psychopathology. Consequently, it may be that only some siblings developed an increased sensitivity to stress as service users, which may not be detectable at the group-level.

Secondary analyses revealed that, similar to exposure to overall LEs, loss, threat, and conflict events modified stress sensitivity in service users. In line with earlier findings reporting associations between the experience of loss and depressive symptoms in youth [16], we found that exposure to loss within the last year increased help-seeking individuals’ negative affect in response to stress. Similarly, conflict events during the past year were associated with higher negative affect in response to stress in service users, while, in siblings, these effects were not found. This contrast between service users and their siblings is particularly interesting as these groups have probably been exposed to comparable levels of several assessed LEs (e.g., parental death, serious persistent quarrels with members within the family). Notably, service users and siblings do not only share genetic risk but also exposure to some socio-environmental risk.

The findings that exposure to LEs did not modify stress sensitivity in siblings and controls or resulted in lower affective reactivity to stress in controls may be partly explained by various protective factors. These may be differentially available in or used by controls and siblings compared to help-seeking individuals. Accumulating evidence suggests that social support, optimism, higher self-esteem, family/neighbourhood cohesion, parental involvement, positive atmosphere at home, low polygenetic risk, and low rumination tendencies contribute to helping individuals in light of ACEs [45–49]. It may be speculated that these processes protect individuals from an increased stress sensitivity by supporting helpful coping strategies and cognitive factors (e.g., greater cognitive flexibility [50]). A recent study demonstrated that psychological flexibility moderates the association between LEs and depressive symptoms and may therefore be considered a “buffer” against unfavourable impacts that LEs have on mental health [51].

Future studies may further explore the proposed transdiagnostic risk mechanism by also considering the role of cognitive factors in individuals’ stress response (e.g., cognitive appraisal [17, 52], aberrant salience, jumping-to-conclusions bias, and theory of mind). Moreover, it is important to investigate whether the effects of ACEs on stress sensitivity accumulate over time (e.g., using cohort designs and, for instance, calculating individuals’ environmental load) [53]. Lastly, exploring the contribution of stress sensitivity in symptom progression and persistence over time by using longitudinal EMA designs may be an important next step. In accordance with prior work that psychiatric symptoms frequently co-occur during developmentally early stages of psychopathology [4], high proportions of comorbid depressive, anxiety, and psychotic symptoms in service users further supports dimensional models of psychopathology [54] as well as transdiagnostic phenotypes, including an extended psychosis spectrum phenotype [5].

## Conclusion

Our results suggest that stress sensitivity may reflect an important risk and resilience mechanism through which LEs negatively impact mental health in help-seeking youth. While we found no unfavourable effects of exposure to LEs on stress sensitivity in controls and siblings, service users appeared to be at greater risk of experiencing elevated stress sensitivity. Targeting sensitivity to stress in daily life with novel mHealth tools (e.g., ecological momentary interventions) by focusing on emotion regulation skills (e.g., mindfulness-based or compassion-focused therapies) may be a promising preventive as well as intervention strategy helping adolescents and young adults with mental health problems [55–57].

## Supplementary Information

Below is the link to the electronic supplementary material.

Supplementary file1 (DOCX 26 KB)

## Data Availability

The data that support the findings of this study are available on reasonable request from the corresponding author C.R.

## Code availability

The code used for statistical analyses are available on reasonable request from the corresponding author C.R.
