# Supplementary material for: Negative life events and stress sensitivity in youth’s daily life: an ecological momentary assessment study
Source: Soc Psychiatry Psychiatr Epidemiol. 2022 Apr 25;57(8):1641–57. doi: 10.1007/s00127-022-02276-0 (PMC9288944; doi:10.1007/s00127-022-02276-0)
Supplement: Supplementary file 1 — Supplementary file1 (DOCX 26 KB) [file 127_2022_2276_MOESM1_ESM.docx]

**Table S1.** Items used in the modified List of Threatening Events (LTE) questionnaire

| **Life event categories** | | |
| --- | --- | --- |
| **Illness** | |  |
|  | LE_001 | “Serious illness, injury or assault to yourself?” |
|  | LE_002 | “Serious illness, injury or assault to a family member or friend?” |
|  | LE_018 | “Hospitalisation or other medical treatments?” |
| **Loss** | |  |
|  | LE_003 | “Death of a parent, partner, or child?” |
|  | LE_004 | “Death of a family member or friend?” |
|  | LE_015 | “Loss or theft of anything of value?” |
| **Interpersonal/**  **Intra-familial conflicts** |  | |
|  | LE_005 | “Break-up with partner (with whom you lived together)/ from parents?” |
|  | LE_006 | “Break-up committed relationship (not living together)/ of a parent?” |
|  | LE_007 | “Divorce/ of parents?” |
|  | LE_008 | “Cheating of spouse (one of the parents)?” |
|  | LE_009 | “Serious persistent problems (quarrel) with members within the family?” |
|  | LE_010 | “A serious problem with a friend, neighbour, or family member?” |
| **Occupational conflicts** | |  |
|  | LE_011 | “Serious problems at school/work (being suspended/ stopped/ fired/ finding no work)?” |
| **Financial problems** | |  |
|  | LE_012 | “Serious financial problems within the family (very serious debt, bankruptcy)?” |
| **Housing problems** | |  |
|  | LE_013 | “Serious housing problems (including homelessness)?” |
| **Legal conflicts** | |  |
|  | LE_014 | “A problem with the police and/or law (violation, court, prison / community service)?” |
| **Threatening/**  **Intrusive incidents** | |  |
|  | LE_016 | “Victim of threats, robbery, or burglary?” |
|  | LE_017 | “Witness to serious threat or another traumatic event?” |
|  | LE_021 | “Involved in a serious accident in which you and/or someone else got hurt seriously?” |
